# Prediction of positive pulmonary nodules based on machine learning algorithm combined with central carbon metabolism data

**DOI:** 10.1007/s00432-024-05610-y

**Published:** 2024-01-25

**Authors:** Jian-jun Liu, Wen-bin Shen, Qi-rong Qin, Jian-wei Li, Xue Li, Meng-yu Liu, Wen-lei Hu, Yue-yang Wu, Fen Huang

**Affiliations:** 1 Department of Epidemiology and Biostatistics, School of Public Health, Anhui Medical University, Hefei, Anhui China; 2 Ma’anshan Center for Disease Control and Prevention, Ma’anshan, Anhui China

**Keywords:** Pulmonary nodule, Predictive model, Central carbon metabolism, Machine learning, SHapley Additive exPlanations

## Abstract

**Background:**

Lung cancer causes a huge disease burden, and early detection of positive pulmonary nodules (PPNs) as an early sign of lung cancer is extremely important for effective intervention. It is necessary to develop PPNs risk recognizer based on machine learning algorithm combined with central carbon metabolomics.

**Methods:**

The study included 2248 participants at high risk for lung cancer from the Ma'anshan Community Lung Cancer Screening cohort. The Least Absolute Shrinkage and Selection Operator (LASSO) was used to screen 18 central carbon-related metabolites in plasma, recursive feature elimination (RFE) was used to select all 42 features, followed by five machine learning algorithms for model development. The performance of the model was evaluated using area under the receiver operator characteristic curve (AUC), accuracy, precision, recall, and F1 scores. In addition, SHapley Additive exPlanations (SHAP) was performed to assess the interpretability of the final selected model and to gain insight into the impact of features on the predicted results.

**Results:**

Finally, the two prediction models based on the random forest (RF) algorithm performed best, with AUC values of 0.87 and 0.83, respectively, better than other models. We found that homogentisic acid, fumaric acid, maleic acid, hippuric acid, gluconic acid, and succinic acid played a significant role in both PPNs prediction model and NPNs vs PPNs model, while 2-oxadipic acid only played a role in the former model and phosphopyruvate only played a role in the NPNs vs PPNs model. This model demonstrates the potential of central carbon metabolism for PPNs risk prediction and identification.

**Conclusion:**

We developed a series of predictive models for PPNs, which can help in the early detection of PPNs and thus reduce the risk of lung cancer.

**Supplementary Information:**

The online version contains supplementary material available at 10.1007/s00432-024-05610-y.

## Introduction

Lung cancer is one of the major malignant tumors in the world, which poses a great threat to human health (Sung et al. 2021). Since lung cancer has no obvious symptoms in the early stage, most patients have developed to the middle and late stages by the time they are detected, and survival rates are greatly reduced (Siegel et al. 2012). Pulmonary nodules (PNs) are an early sign of lung cancer. Early detection and intervention of PNs can significantly improve the prognosis of patients with lung cancer (Ost and Fein 2000).

PNs are a focal, circular, dense solid or solid pulmonary shadow with a diameter of 3 cm or less, without atelectasis, hilar lymph node enlargement, or pleural effusion (Swensen et al. 2002). Studies have confirmed that PNs of different sizes and properties have different abilities to develop into lung cancer. Compared with solid nodules, subsolid nodules are more prone to malignant changes, and their potential malignancy rate is significantly higher than solid nodules (Carreras and Gorini 2017). The larger the PNs are, the higher the likelihood of later deterioration, the study found (Mazzone and Lam 2022; Vachani et al. 2022). The prevalence of lung cancer is 0–1% in 6 mm nodules, 1–2% in 6–8 mm nodules, and approximately 10% in 8 mm nodules. Therefore, it is necessary for early detection, diagnosis and intervention of PNs.

Research on the factors influencing PNs is limited and focuses on traditional demographic factors such as smoking, history of lung disease, occupational exposure, and genetic factors (Peng et al. 2010; Ruparel et al. 2016). It has been suggested that metabolites in the body may also be involved in the development of nodules (Abooshahab et al. 2020; Gao et al. 2013). A plasma metabolomics and lipidomics study involving 1160 participants showed that metabolites associated with arginine and proline metabolism were elevated in benign isolated PNs, while metabolites associated with fatty acid and acylcarnitine metabolism were decreased (Zhou et al. 2022). These studies suggest that amino acid metabolism and lipid metabolism play an important role in the occurrence and development of PNs. Abnormal cell proliferation requires the consumption of additional energy and biosynthetic precursors relative to normal cells (Hensley et al. 2016). Central carbon metabolism, also known as energy metabolism, is the core pathway of cell metabolism, which is closely related to abnormal cell proliferation. Previous studies have shown that central carbon metabolism plays an important role in the occurrence and development of lung diseases (Kim et al. 2022; Weckerle et al. 2022). Central carbon-related metabolites are expected to be effective predictors of PNs.

In the medical field, the powerful data processing and computing power of machine learning is very popular. Compared with traditional statistical analysis methods, machine learning algorithms not only have fewer restrictive assumptions about data distribution, but also can identify interaction effects and relationships among relevant factors (Burgos and Colliot 2020). At the same time, the machine learning algorithm reduces the influence of sample error by randomly selecting samples several times before model training, which makes the model results more reliable. Therefore, it is suitable for processing high-dimensional complex data and identifying and predicting diseases. Support vector machine (SVM) has been used to achieve accurate identification of esophageal squamous cell carcinoma (Yuan et al. 2021). Michael K. Gould et al. (2021) showed that the prediction model constructed using ML was better than previous models. Machine learning has also excelled in predicting lung cancer risk (Huang et al. 2022; Li et al. 2022). At the same time, in order to better explain the machine learning model, we introduce SHapley Additive exPlanations (SHAP). It is now widely used to build interpretable machine learning frameworks and interpret their predictions (Ballester et al. 2021; Fan et al. 2022; Yang et al. 2021).

In order to investigate the influence of central carbon metabolites on the occurrence and development of positive pulmonary nodules, and then achieve the purpose of accurate identification of positive pulmonary nodules. In this study, we attempted to construct a predictive model using central carbon metabolite data to predict the risk of detecting PPNs in people at high risk for lung cancer.

## Materials and methods

### Study population

This study is based on the Ma’anshan Lung Cancer Screening Cohort (MALSC), a prospective cohort that has been described in previous study (Zhu et al. 2023). A total of 10,038 community populations were enrolled in the MALSC at the beginning of baseline survey. After risk assessment, 2289 high-risk individuals were screened with LDCT imaging and tested for central carbon metabolites in plasma. Then, there were 41 subjects missing important information such as central carbon metabolites and imaging findings. After that, the data of 2248 subjects were included in this analysis. The specific process of this study is shown in Fig S1.

This study was approved by the Ethics Committee of Ma’anshan Center for Disease Control and Prevention (Approval No.2020001), and all participants signed informed consent prior to the study.

### Data collection

Due to the different nature and size of pulmonary nodules with different risk of developing into lung cancer, in order to stratify the risk of different pulmonary nodules, solid or partially solid nodules with diameter ≥ 5 mm, or non-solid nodules with diameter ≥ 8 mm and endotracheal nodules detected were defined as positive pulmonary nodules (PPNs) in this study according to the national Cancer Screening guidelines. If the nodule diameter is smaller than this standard and no tracheal nodule is detected, it is defined as negative pulmonary nodule (NPNs) (Zhou et al. 2018).

From the questionnaire, we collected demographic characteristics (age, sex, annual household income and education), behavioral habits (smoking status, alcohol consumption status, and exercise status), personal disease history and family history. At the same time, we also collected basic clinical laboratory test data from hospitals.

A total of 2248 serum samples were analyzed by central carbon metabolomics. As there are few metabolomic studies on PNs at present, we also considered those related to lung cancer while reviewing the metabolomic literatures related to PNs. We identified candidate metabolites through literature review and non-targeted metabolomics experiments. An exploratory preliminary trial was then conducted in a small sample of findings that included 16 lung cancers and 32 controls. Finally, after reviewing the literature and exploring a small sample, we determined to use high-performance ion chromatography-mass spectrometry (HPIC-MS) to target the detection of 18 central carbon-related metabolites.

### Statistical analysis

Continuous variables have been expressed as mean ± SD or medians with IQRs, and compared using the Wilcoxon test when data were not normally distributed. Categorical variables have been reported as numbers and percentages, and compared using a Chi-square test or Fisher exact test. All analyses were conducted using Python, version 3.9.5.

### Feature selection

First, we removed metabolites with a deletion rate of ≥ 30%. For metabolites with less than 30% of the missing metabolite, the lowest detectable limit for that substance is used. Spearman rank correlation method was used to calculate the correlation between the concentrations of 18 metabolites. Then, the Least Absolute Shrinkage and Selection Operator (LASSO) penalty regression was used to screen the central carbon-related metabolites. In short, LASSO is a regression contraction and selection method that imposes a penalty on the component regression coefficient and is suitable for working with complex multicollinearity data (Dai et al. 2016).

Recursive feature elimination (RFE) was applied feature selection with a tenfold cross-validation during the elimination process. The ultimate determination of the selected features was made by taking into account both the number of variables and the area under the receiver operating characteristic curve (AUC).

### Model training

The objective of this study is to construct a prediction model of PPNs based on machine learning algorithm combined with central carbon metabolism data. We partitioned the dataset into training and testing sets, using an 80% to 20% split. The training set is used for model development, in which grid search is used for hyperparameter tuning and optimal threshold determination. Adhere to the testing set, the model is not seen in training, only used in performance evaluation. Regarding the algorithm used in the development of prediction models, we chose five machine learning algorithms, including random forest (RF), XGBoost, SVM, naive Bayes (NB) and decision tree (DT), to construct models based on the results of the feature selection. We chose these five learning algorithms because they were recommended in a multidisciplinary machine learning guide (Luo et al. 2016).

In order to better predict and identify PPNs, in this study, we developed two PPNs prediction models using the same training method. The first model predicted PPNs in all lung cancer high-risk groups, and the other model was developed only in PNs, with the purpose of distinguishing PPNs and NPNs.

### Model introduction

#### Decision tree

The decision tree proposed in this study is based on a binary tree algorithm-classification regression tree (CART). Gini coefficient is used as the partitioning standard. The larger the Gini coefficient, the higher the uncertainty of the data is.

#### Random forest

Random forest is an integrated algorithm that can fuse multiple decision trees together. In RF, each decision tree is equivalent to a classifier with lower strength. When all decision trees are successfully constructed, the random forest can summarize the voting classification results made by each decision tree to get the final result.

#### SVM

SVM algorithm would learn by giving inputs of label-data statistics to build a binary discriminative classifier. It defines a separating hyperplane or finds the “maximum-margin” to discriminate between groups. The weight representing importance in classification for all features was generated.

#### Naive Bayes

Naive Bayes algorithm is based on simplified Bayes algorithm, and its simplicity lies in its very simple ideological foundation. In the case of a given target, the properties are assumed to be mutually conditional independent. In the given training sample, based on the assumed joint probability distribution of input and output, the output of the maximum posterior probability is obtained on the basis of the model. The implementation of naive Bayes algorithm is very simple and has good learning and prediction ability.

#### XGBoost

XGBoost is an excellent ensemble learning model, the main idea is to take decision tree as the base learner, and then build them in parallel based on boosting framework, and finally integrate them into a strong learner with higher accuracy. When fitting the model, XGBoost first calculates the predicted value of each tree, then carries out the second-order Taylor expansion on the residual of the previous tree, and has its own regularization term, which can effectively prevent over-fitting and improve the generalization performance of the model. Finally, the results of multiple decision trees are weighted and averaged.

### Measuring model performance

The differential ability of several models to predict positive pulmonary nodules from negative pulmonary nodules and normal subjects was evaluated and compared using receiver operator characteristic (ROC) curve analysis. AUC greater than 0.5 indicates better predictive performance of the models. Accuracy, precision, recall, and F1 scores were also used to evaluate the performance and generalization of each of models, where accuracy represents the proportion of all samples correctly classified by the model and is used to measure the accuracy of the overall prediction, but accuracy may not be the most appropriate evaluation criteria in unbalanced categories or cost sensitivity; precision, which is the proportion of true positives among predicted positives, measures how correct the model is in the case of predictions in the positive category, and it is important in medical diagnosis where you want to avoid misdiagnosis; recall rate refers to the proportion of true positives among all positive instances, and measures the degree to which the model captures true positive cases, which is even more important in cases such as cancer detection where the hope is to minimize missed diagnoses; F1 score is the harmonic average of precision and recall, and is a comprehensive indicator for situations where there is a balance between accuracy and recall.

### Model interpretability

In order to enhance the explainability of the model, the method of SHapley Additive exPlanations (SHAP) is introduced in this study. SHAP, which originated from game theory, can provide an explanation of the model’s output, so as to answer the question of how much a particular feature contributes to the overall model's predictive effectiveness (Yanamala et al. 2021). The resulting SHAP values quantify the direction and magnitude of the feature's influence on a given prediction. The greater the absolute SHAP value of the feature is, the greater its influence on the prediction. The direction of the SHAP value in the diagram indicates whether the feature is influential or indicative on the negative or positive class.

## Results

### Descriptive statistics

Of the 2248 participants, 284 had PPNs and 537 had NPNs. Compared to normal and NPNs, PPNs were older (64.5 ± 6.2), more likely to be male, ever smokers, current alcohol consumer and those exposed to indoor incense. The general demographic characteristics of the participants are detailed in Table 1. Table S1 shows that the basic clinical indicators were basically similar among the three groups, and total cholesterol in the PPNs group was lower than that in the normal and NPNs groups.

**Table 1:** Demographic characteristics of high-risk groups for lung cancer

Variables	Normal (*n* = 1427)	Negative (*n* = 537)	Positive (*n* = 284)
Age (years, mean ± SD)	63.0 ± 6.7	63.2 ± 6.7	64.5 ± 6.2
Sex (*n*, %)			
Male	987 (63.7)	353 (22.8)	210 (13.5)
Female	440 (63.0)	184 (26.4)	74 (10.6)
Education level (*n*, %)			
Elementary school or below	451 (64.1)	152 (21.6)	101 (14.3)
Middle school	563 (62.3)	234 (25.9)	107 (11.8)
High school/technical school	328 (64.8)	116 (22.9)	62 (12.3)
College or above	85 (63.4)	35 (26.1)	14 (10.4)
Household income (RMB/per year) (*n*, %)			
< 30,000	151 (63.7)	52 (21.9)	34 (14.3)
30,000 ~	449 (64.4)	158 (22.7)	90 (12.9)
60,000 ~	507 (61.5)	199 (24.2)	118 (14.3)
> 90,000	320 (65.3)	128 (26.1)	42 (8.6)
Asbestos exposure (*n*, %)			
Yes	1359 (63.6)	507 (23.7)	12 (10.9)
No	68 (61.8)	30 (27.3)	272 (12.7)
Smoking status (*n*, %)			
Never	798 (64.0)	302 (24.2)	146 (11.7)
Ever	629 (62.8)	235 (23.5)	138 (13.8)
Drinking status (*n*, %)			
Never	756 (63.6)	303 (25.4)	135 (11.3)
Current	540 (62.6)	193 (22.4)	129 (15.0)
Ever	131 (68.2)	41 (21.4)	20 (10.4)
Indoor incense burning (*n*, %)			
Yes	1307 (64.2)	486 (23.9)	42 (19.7)
No	120 (56.3)	51 (23.9)	242 (11.9)
History of pulmonary disease (*n*, %)			
Yes	1272 (64.0)	469 (23.6)	37 (14.2)
No	155 (59.6)	68 (26.2)	247 (12.4)
Family history of cancer (*n*, %)			
Yes	739 (63.1)	279 (23.8)	130 (12.1)
No	688 (63.9)	258 (24.0)	154 (13.1)

The analysis of 18 plasma central carbon-related metabolites of participants showed that plasma concentrations of 2-ketoglutaric acid, 3-hydroxybutyric acid, gluconic acid, phosphoenolpyruvic acid, glyceric acid, succinic acid, hippuric acid, citric acid, malic acid, L-lactic acid, cis-aconite acid and isocitric acid in PPNs were significantly higher than those in no pulmonary nodules and negative pulmonary nodules groups (*P* < 0.001). Plasma concentrations of 2-oxadipic acid, homogentisic acid, maleic acid and ortic acid in pulmonary nodule group were significantly lower than those in non-pulmonary nodule group (*P* < 0.05). There was no significant difference in the concentration of fumaric acid and glucaric acid between groups (*P* > 0.05), as shown in Table S2. Spearman correlation coefficients among these metabolites ranged from – 0.14 to 0.71 (Fig S2).

### PPNs risk prediction model in high-risk lung cancer population

Based on the results of LASSO regression screening, a total of 7 central carbon metabolites were selected for subsequent prediction model construction. They are homogentisic acid, 2-oxadipic acid, fumaric acid, maleic acid, succinic acid, gluconic acid, hippuric acid (Fig S3). To develop the predictive model, all features were screened using the RFE method. The RFE results are shown in Table 2. Considering the simplicity and Accuracy of the prediction model, we finally chose a model containing 10 features (Accuracy = 0.9163, Kappa = 0.5762). The features included in the model are as follows homogentisic acid, 2-oxadipic acid, fumaric acid, maleic acid, succinic acid, gluconic acid, hippuric acid, Monocyte ratio (MR), basophil count (BLC) and triglyceride (TG). The performance of each model was evaluated on the test set.

**Table 2:** Recursive feature elimination coupled with the random forest is employed for selecting features

	Number of features	AUC	Accuracy	Kappa	Accuracy SD	Kappa SD
PPNs risk prediction model in high-risk lung cancer population	42	0.8499	0.9140	0.5612	0.0353	0.2848
	22	0.8452	0.9118	0.5605	0.0367	0.2930
	10	0.8346	0.9163	0.5762	0.0353	0.2701
	5	0.8257	0.9140	0.5683	0.0349	0.2566
PPNs versus NPNs	42	0.8365	0.7963	0.5355	0.0780	0.2194
	22	0.8462	0.8086	0.5515	0.0775	0.2165
	10	0.8372	0.8025	0.5434	0.0826	0.2274
	5	0.8122	0.7901	0.5236	0.0885	0.2410

**Fig. 1:**
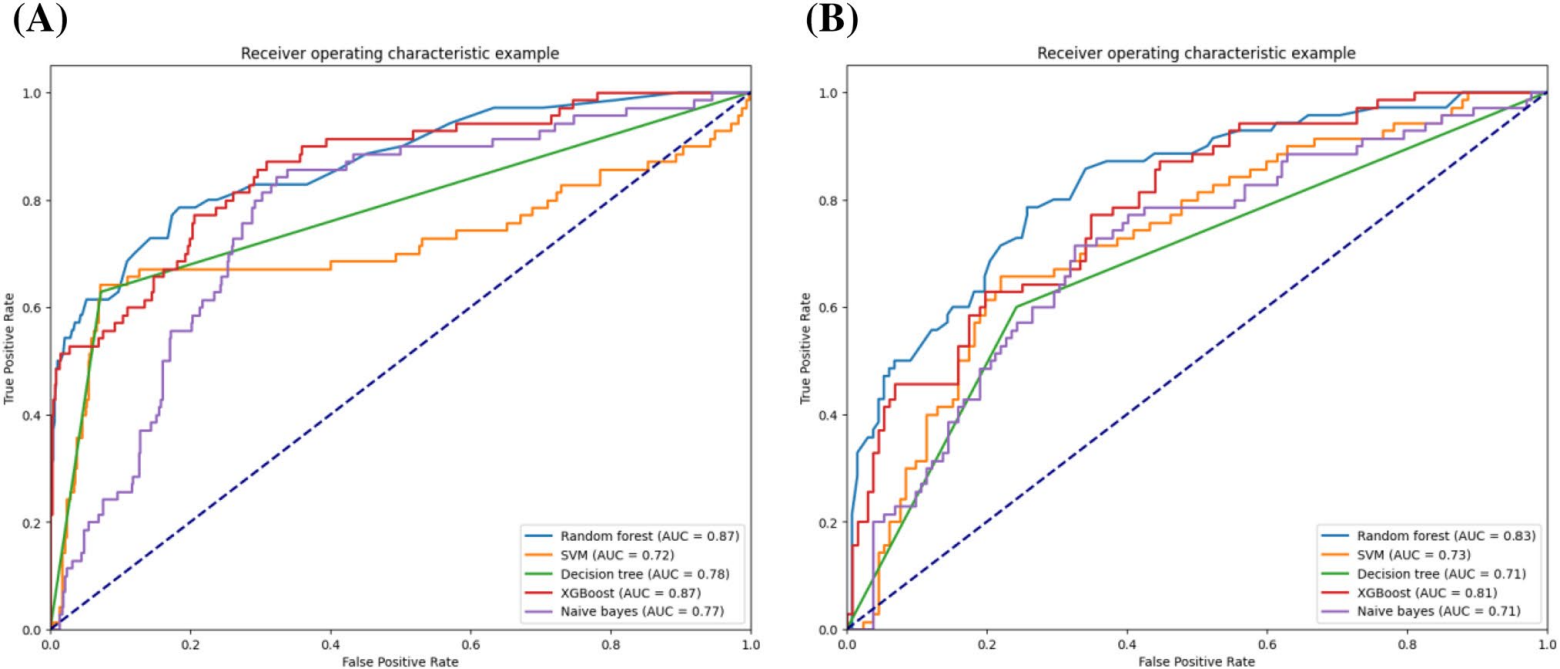
ROC curves of positive pulmonary nodules prediction models constructed by different machine learning algorithms in the test set. **A** PPNs risk prediction model in high-risk lung cancer population. **B** PPNs versus NPNs. XGBoost: Extreme Gradient Boosting; SVM: support vector machine

The ROC curve analysis indicated that RF and XGBoost attained the highest predictive performance with an AUC of 0.87. The AUC values of DT, NB and SVM are 0.78, 0.77 and 0.72, respectively, showing a relatively worse performance (Fig. 1A). The performance evaluation indexes of each model, such as Accuracy, Recall, Precision and F1 score, are shown in Table 3. RF had the best performance, with an accuracy of 0.93, comparable to XGBoost, followed by DT, SVM and NB, which were 0.88, 0.87 and 0.84, respectively.

**Table 3:** Performance indicators of different machine learning models for predicting PPNs

	Model	AUC	ACC	Precision	Recall	F1 score
PPNs risk prediction model in high-risk lung cancer population	Random forest	0.87	0.93	0.92	0.93	0.92
	XGBoost	0.87	0.92	0.92	0.93	0.92
	Decision tree	0.78	0.88	0.88	0.88	0.88
	Naive Bayes	0.77	0.84	0.82	0.84	0.83
	SVM	0.72	0.87	0.83	0.87	0.83
PPNs versus NPNs	Random forest	0.83	0.86	0.92	0.93	0.92
	XGBoost	0.81	0.84	0.89	0.93	0.91
	Decision tree	0.71	0.67	0.74	0.88	0.81
	Naive Bayes	0.71	0.58	0.64	0.84	0.72
	SVM	0.73	0.61	0.67	0.87	0.76

*XGBoost* Extreme Gradient Boosting, *SVM* support vector machine

### PPNs versus NPNs

To further distinguish between negative and positive pulmonary nodules, we trained another five machine learning models in PNs in the same way. When LASSO was used to screen the specific central carbon metabolites between negative and positive pulmonary nodules, seven metabolites were selected, namely homogentisic acid, phosphoenolpyruvic acid, fumaric acid, maleic acid, succinic acid, gluconic acid, and hippuric acid (Fig S4). As before, the results of recursive feature elimination feature selection are shown in Table 2**.** The features included in the model are as follows homogentisic acid, phosphoenolpyruvic acid, fumaric acid, maleic acid, succinic acid, gluconic acid, hippuric acid, low-density lipoprotein (LDL), high-density lipoprotein (HDL) and mean corpuscular volume (MCV).

The ROC curve analysis indicated that RF attained the highest predictive performance with an AUC of 0.83, closely trailed by XGBoost with an AUC of 0.81. SVM had relatively poor performance (AUC = 0.73), and DT and NB models both had the lowest AUC of 0.71 (Fig. 1B).

### Model interpretability

**Fig. 2:**
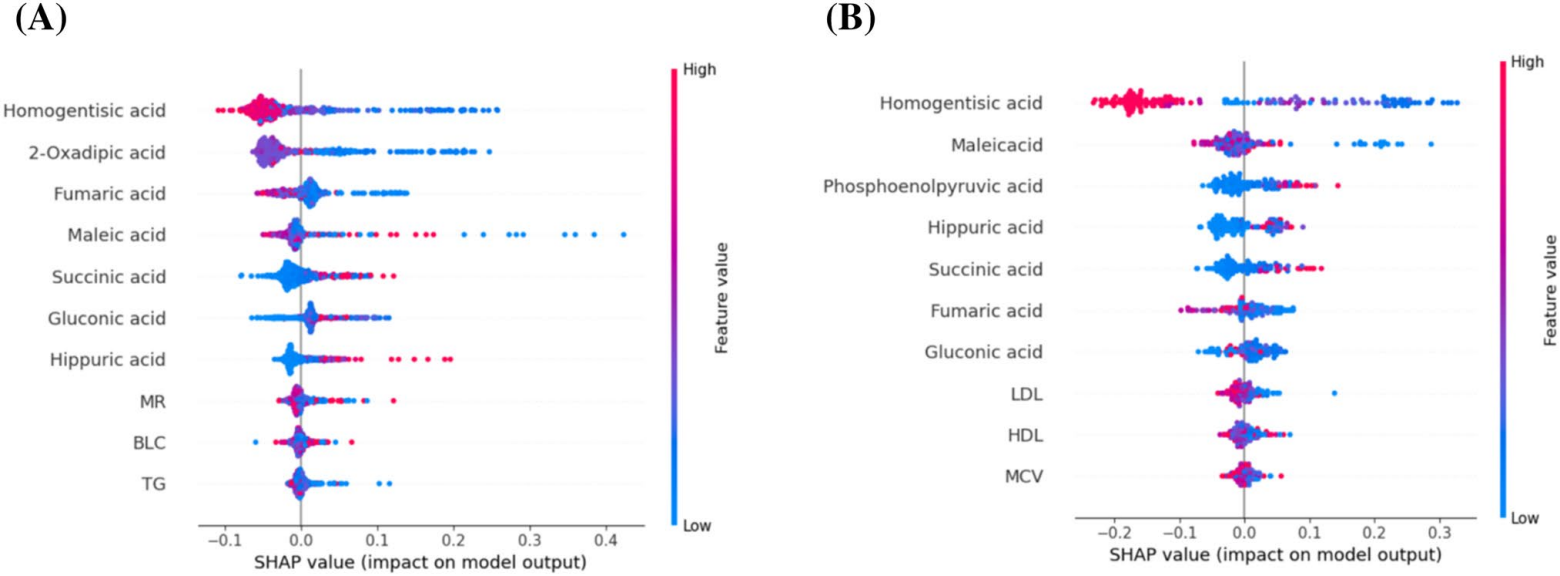
SHapley Additive exPlanations (SHAP) summary for **A** PPNs risk prediction model in high-risk lung cancer population. **B** PPNs versus NPNs. The summary plot combines feature importance with feature effects. The features on the y-axis are ordered according to their importance. Each point on the summary plot is a SHapley value for a feature and an instance in the dataset. The position of each point on the x-axis shows the impact that feature has on the classification model’s prediction for a given instance. The color represents the high (red) to low (blue) values of the feature

The SHAP method was used to gain insights into the importance of features and interpret the predictions of the RF model for the risk of PPNs in high-risk lung cancer population. Through SHAP analysis (Fig. 2A), we found that the top 7 features that contributed most to the prediction of PPNs in the lung cancer high-risk population were central carbon-related metabolites. Intuitively, higher succinic acid, gluconic acid and hippuric acid lead to a greater risk of PPNs, while higher homogentisic acid, 2-oxadipic acid, fumaric acid and maleic acid leads to a lower risk of PPNs. Similarly, in the PPNs versus NPNs models, higher phosphoenolpyruvic acid, succinic acid, gluconic acid and hippuric acid led to a greater risk of PPNs, while higher homogentisic acid, fumaric acid and maleic acid led to a lower risk of PPNs (Fig. 2B).

**Fig. 3:**
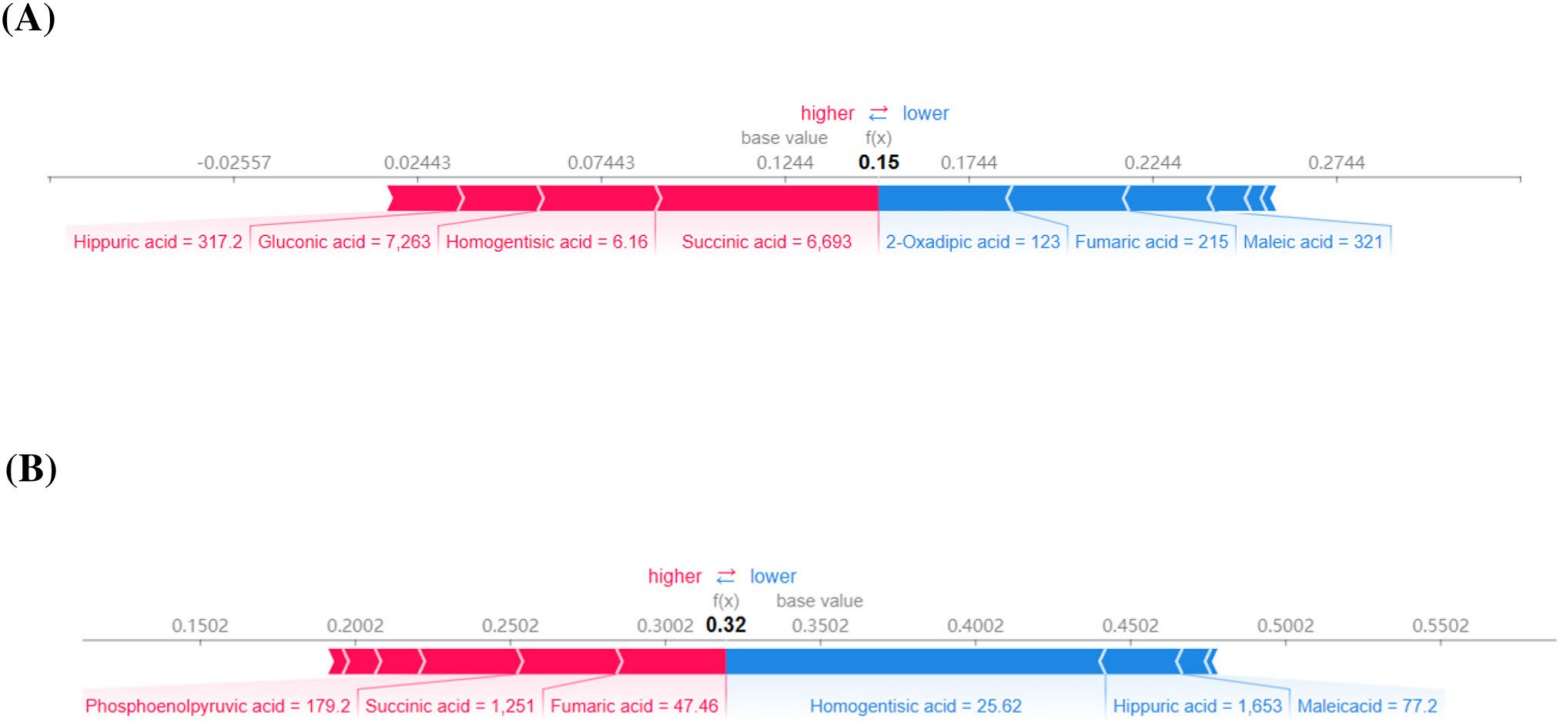
SHapley Additive exPlanations (SHAP) force plots. **A** PPNs risk prediction model in high-risk lung cancer population. **B** PPNs versus NPNs

The SHAP diagram shown in Fig. 3 is intended to provide an explanation for the individual predictions made by our model. Figure 3a shows an encounter that was correctly classified as PPNs, with multiple central carbon metabolites having the greatest impact on model output. The selected individuals had succinic acid of 6693 ng/mL, homogentisic acid of 6.16 ng/mL, gluconic acid of 7263 ng/mL and hippuric acid of 317.2 ng/mL, which increased the predicted risk of PPNs. Conversely, 2-oxadipic acid of 123 ng/mL, fumaric acid of 215 ng/mL, maleic acid of 321 ng/mL, platelet of 247 10^9^/L, monocyte ratio of 6% and triglyceride of 2.14 mmol/L reduced the predicted risk of PPNs. The final model output value is 0.15, which is greater than the base value 0.1244, so it is correctly classified as PPNs. Figure 3b illustrates an encounter that was correctly classified as NPNs. The selected individuals had homogentisic acid of 25.62 ng/mL, hippuric acid of 1653 ng/mL, maleic acid of 77.2 ng/mL and HDL of 1.75 mmol/L, which reduced the predicted risk of PPNs. This offset the increased risk associated with fumaric acid of 47.46 ng/mL, succinic acid of 1251 ng/mL, phosphoenolpyruvic acid of 179.2 ng/mL and mean corpuscular volume of 97 fL. Finally, this individual was correctly classified as NPNs. With these interpretability methods, we are able to clearly determine the reasons for the model’s output and ensure they can be scrutinized.

## Discussion

The aim of this study was to develop a series of well-performing machine learning-based models for assessing the risk of detecting PPNs in people at high risk for lung cancer. To our knowledge, this may be the first machine learning model built based on central carbon metabolism to predict PPNs in people at high risk of lung cancer. This study fills an important gap in the knowledge of the concentration levels of various plasma central carbon metabolites and their relationship with PPNs in lung cancer high-risk populations. Using LASSO regression, we identified the most important metabolites in the mixture and used them for subsequent modeling. The results show that central carbon-related metabolites have a surprising predictive power for PPNs risk in people at high risk for lung cancer. By comparing different algorithms for predicting PPNs in people at high risk of lung cancer, we propose two RF models based on demographic factors, basic clinical examination indicators, and blood levels of central carbon-related metabolites. Similar to our results, random forest also performed best in a study of survival predictions for head and neck cancer (Salmanpour et al. 2023). The model achieves good performance, with AUC values of 0.87 and 0.83, respectively. These results were internally verified to show that the results were not found by chance, which is acceptable reliability. In terms of accuracy, the two models in this study (ACC = 0.93 and 0.86) are superior to the integrated model based solely on image information (ACC = 0.790) of Huang et al., which is similar to the study of Heydarheydari et al. (ACC = 0.9403 ± 0.0444) (Huang et al. 2022; Heydarheydari et al. 2023).

Referring to previous studies (Hosseinzadeh et al. 2023; Rezaeijo et al. 2022), we trained the model in metabolite-only datasets, demographic characteristics and clinical general detection indicators datasets respectively. The results showed that the performance of the metabolite-only model was similar to the model in this study, and was much better than the model with demographic characteristics and clinical general indicators, which also showed the good risk prediction ability of central carbon-related metabolites for PPNs (Fig S5). The model of demographic characteristics plus clinical general detection indicators did not perform well, because the study population was assessed based on the high-risk criteria for lung cancer screening, and the distribution of demographic characteristics such as smoking was similar among the groups.

To enhance the interpretability of the model, we employed SHAP values, which provided both global and local explanations for the model’s predictions. The global explanations highlighted the average contribution of each feature across the entire dataset, while the local explanations demonstrated the influence of each feature on individual sample predictions (Martin et al. 2023). This aspect of interpretability is valuable for understanding the underlying factors driving the predictions of the model and can be utilized for clinical decision-making and interventions.

Feature-importance analysis and overall interpretation of SHAP indicate that central carbon-related metabolites are important predictors of PPNs risk in high-risk lung cancer populations. For PPNs risk prediction model and PPNs recognition model, homogentisic acid, phosphoenolpyruvic acid, fumaric acid, maleic acid, succinic acid, gluconic acid and hippuric acid play an important role in both models. Succinic acid and fumaric acid are two metabolites in the TCA cycle, which may affect the occurrence and development of PPNs through energy metabolism and cellular hypoxia (Beloborodova et al. 2019; Lee et al. 2020). Homogentisic acid and maleic acid are upstream substances of fumaric acid, which can affect PPNs through fumaric acid. Hippuric acid is a metabolomic marker of gut microbiota diversity (Pallister et al. 2017). Studies have found that gut microbiota can influence the development of lung diseases through the gut-lung axis (Zhao et al. 2021). Our study found that the level of hippuric acid was higher in PPNs group, and the imbalance of intestinal flora may play a role in the development of PPNs. A study found that gluconic acid and markers of oxidative stress, we speculate that gluconic acid affects the occurrence and development of PPNs through oxidative stress, and the specific process needs more research to explore (Feng et al. 2018). 2-oxadipic acid is present only in PPNs prediction models of lung cancer high-risk populations and is a metabolite of the breakdown of essential amino acids lysine and tryptophan. The tryptophan pathway is thought to play a key role in inflammation and immune regulation (Shibata et al. 2011). Our study found that PPNs participants had lower levels of 2-oxadipic acid concentration than controls, suggesting that the lysine and tryptophan catabolic pathways may be involved in the occurrence of PPNs. Similarly, phosphoenolpyruvic acid, which only plays a role in PPNs recognition models, is an intermediate product of sugar degradation, a process that has been shown to be associated with lung fibrosis as well as smooth muscle cell proliferation in COPD.

Our research has several advantages. First, to our knowledge, this may be the first study to use a machine learning algorithm combined with central carbon metabolism to predict PPNs in people at high risk for lung cancer. The addition of central carbon metabolism allows us to more accurately assess the risk of PPNs, so as to detect, diagnose and intervene in PPNs as early as possible to reduce the harm of lung cancer. Second, we conducted a comprehensive comparison of five commonly used machine learning algorithms and determined that RF models performed best in predicting PPNs risk. This comparison not only helps to select the most suitable model for this study, but also provides valuable guidance for future research and practical application. In addition, this study separately established the prediction model suitable for different situations. It can not only predict the likelihood of detecting PPNs in lung cancer high-risk groups, but also assess the risk of converting PNs to PPNs. Finally, we use SHAP values to provide global and local interpretations of our predictive model. Global interpretations highlight the average contribution of each feature across the entire dataset, while local interpretations show the impact of each feature on individual sample predictions. SHAP attempts can visually assess the likelihood of PPNs in people at high risk of lung cancer, and this information can help researchers develop appropriate intervention strategies to reduce the occurrence of PPNs.

There are also some limitations to this study. First, the cross-sectional design limits our ability to infer a causal relationship between plasma metabolite levels and PPNs. Long-term follow-up studies are necessary to refine our model. Second, in recent years, central carbon metabolism has gradually become a hot research direction, but few relevant tests have been carried out in lung cancer screening. Due to the lack of variables in other studies, only internal verification was carried out in this study. In the follow-up study, it is necessary to find a suitable independent cohort for external verification, so as to better evaluate the performance of the model.

## Conclusion

In this study, we developed a series of predictive models based on machine learning algorithms combined with central carbon metabolism to identify risk factors for developing PPNs in people at high risk for lung cancer. The model showed good performance and provided explainable insights that could lead to early detection of PPNs and thus reduce the risk of lung cancer.

## Supplementary Information

Below is the link to the electronic supplementary material.

Supplementary file1 (PDF 1246 KB)

## Data Availability

The dataset used and analyzed during the current study are available from the corresponding author upon reasonable request.

## Abbreviations

AUC: Area under the curve
DT: Decision tree
HPIC-MS: High-performance ion chromatography–mass spectrometry
LASSO: Least absolute shrinkage and selection operator
NB: Naive Bayes
NPNs: Negative pulmonary nodules
PNs: Pulmonary nodules
PPNs: Positive pulmonary nodules
RF: Random forest
SVM: Support vector machine
SHAP: SHapley Additive exPlanations
