# Supplementary material for: Prediction of positive pulmonary nodules based on machine learning algorithm combined with central carbon metabolism data
Source: J Cancer Res Clin Oncol. 2024 Jan 25;150(2):33. doi: 10.1007/s00432-024-05610-y (PMC10811045; doi:10.1007/s00432-024-05610-y)
Supplement: Supplementary file 1 — Supplementary file1 (PDF 1246 KB) [file 432_2024_5610_MOESM1_ESM.pdf]

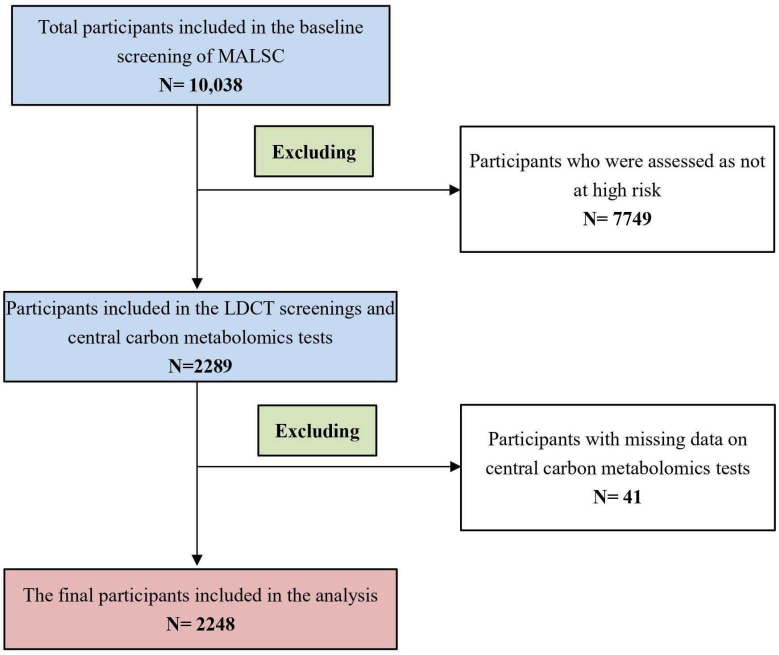


**Fig. S1.** Flow chart for the exclusion and inclusion of research participants.

**Table S1** Distribution of basic clinical indicators in different PNs groups

| | Normal(n=1427) | Negative(n=537) | Positive (n=284) |
|---|---|---|---|
| RBC($10^{12}$/L, mean ± SD) | 4.83 ± 0.5 | 4.85 ± 0.5 | 4.81 ± 0.4 |
| WBC($10^9$/L) | 6.51(5.50,7.60) | 6.60(5.64,7.57) | 6.70(5.72,7.67) |
| Hemoglobin(g/L) | 146.00(136.00,156.00) | 146.00(136.00,156.00) | 145.00(137.00,154.00) |
| Average hemoglobin concentration(g/L) | 319.00(313.00,324.00) | 319.00(313.00,324.00) | 319.00(314.00,325.00) |
| Platelet($10^9$/L) | 180.00(147.00,214.00) | 182.00(143.25,220.00) | 183.00(146.00,215.00) |
| Platelet volume | 0.18(0.15,0.20) | 0.18(0.15,0.21) | 0.18(0.16,0.21) |
| NEUT($10^9$/L) | 3.69(3.02,4.46) | 3.74(3.10,4.45) | 3.82(3.12,4.66) |
| MNC($10^9$/L) | 0.40(0.30,0.46) | 0.40(0.30,0.50) | 0.40(0.30,0.50) |
| Lcteric,n(%) | | | |
| - | 1220(63.6) | 454(23.7) | 243(12.7) |
| + | 207(62.7) | 83(25.2) | 40(12.1) |
| ++ | 0(0.0) | 0(0.0) | 1(100.0) |
| Basophil count($10^9$/L) | 0.30(0.20,0.40) | 0.30(0.20,0.48) | 0.30(0.20,0.50) |
| Eosinophil count($10^9$/L) | 0.14(0.09,0.22) | 0.14(0.09,0.22) | 0.15(0.10,0.26) |
| Lymphocyte count($10^9$/L) | 1.96(1.60,2.40) | 1.97(1.60,2.40) | 2.00(1.60,2.40) |
| Platelet distribution width(%) | 63.30(58.00,68.43) | 63.40(57.90,68.38) | 64.55(58.10,69.20) |
| Lipoblood index,n(%) | | | |
| - | 1371(63.3) | 519(24.0) | 275(12.7) |
| + | 45(65.2) | 15(21.7) | 9(13.0) |
| ++ | 7(77.8) | 2(22.2) | 0(0.0) |
| +++ | 3(75.0) | 1(25.0) | 0(0.0) |
| ++++ | 1(100.0) | 1(100.0) | 0(0.0) |
| Mean platelet volume(fL) | 9.70(8.78,10.90) | 9.70(8.80,10.90) | 9.80(8.90,11.28) |
| Average hemoglobin volume(pg) | 30.30(29.30,31.20) | 30.20(29.33,31.20) | 30.20(29.40,31.10) |
| Mean corpuscular volume(fL) | 94.70(91.90,97.50) | 94.70(91.90,97.50) | 94.50(92.00,97.50) |
| Hemolytic index,n(%) | | | |
| - | 1417(63.6) | 531(23.8) | 280(12.6) |
| + | 9(50.0) | 5(27.8) | 4(22.2) |
| ++ | 0(0.0) | 1(100.0) | 0(0.0) |
| +++ | 1(100.0) | 0(0.0) | 0(0.0) |
| Neutrophil ratio(%, mean ± SD) | 57.5 ± 8.0 | 57.9 ± 7.9 | 58.0 ± 8.0 |
| Lymphocyte ratio(%, mean ± SD) | 30.9 ± 7.5 | 30.6 ± 7.1 | 30.2 ± 7.3 |
| Hematokrit(L/L) | 0.46(0.43,0.49) | 0.46(0.43,0.48) | 0.45(0.43,0.48) |
| Basophil ratio(%) | 0.50(0.40,0.60) | 0.50(0.40,0.60) | 0.50(0.40,0.70) |
| Eosinophilic ratio(%) | 2.20(1.40,3.50) | 2.20(1.40,3.50) | 2.30(1.50,3.60) |
| Monocyte ratio(%) | 5.60(4.70,6.60) | 5.60(4.70,6.60) | 5.60(4.80,6.70) |
| HDL(mmol/L) | 1.34(1.14,1.58) | 1.34(1.14,1.58) | 1.30(1.11,1.51) |
| LDL(mmol/L) | 2.84(2.34,3.41) | 2.84(2.34,3.41) | 2.79(2.27,3.41) |
| Total cholesterol(mmol/L)* | 4.60(4.03,5.19) | 4.60(4.03,5.19) | 4.51(3.81,5.12) |
| Triglyceride(mmol/L) | 1.47(1.06,2.10) | 1.46(1.06,2.09) | 1.43(1.02,1.97) |
| Blood glucose(mmol/L) | 5.06(4.63,5.77) | 5.06(4.63,5.77) | 5.04(4.64,5.91) |

**Table S2** Distribution of plasma central carbon metabolites in different PNs groups (ng/mL)

| | Normal(n=1427) | Negative(n=537) | Positive (n=284) | P-value* |
|---|---|---|---|---|
| 2-Ketoglutaric acid | 2187.5(1514.0,3570.4) | 2199.6(1581.6,3627.4) | 2520.3(1792.5,4700.6) | <0.001 |
| 2-Oxadipic acid | 112.7(107.9,120.3) | 112.4(106.9,120.8) | 75.8(64.8,113.4) | <0.001 |
| 3-Hydroxybutyric acid | 8090.7(4964.1,17194.6) | 8830.1(5625.0,16705.0) | 10833.3(5772.8,26674.2) | <0.001 |
| Gluconic acid | 978.1 (541.0,1397.6) | 1032.7 (538.3,1497.4) | 1093.0(773.5,1747.4) | <0.001 |
| Phosphoenolpyruvic acid | 141.8 (131.6,174.1) | 143.4 (131.3,176.7) | 175.6 (133.1,189.9) | <0.001 |
| Fumaric acid | 76.1(52.8,124.1) | 82.0(53.9,125.9) | 62.5 (51.3,123.2) | 0.096 |
| Glyceric acid | 837.6 (627.3,1206.1) | 860.2 (649.0,1192.0) | 944.9(649.3,1247.1) | <0.001 |
| Succinic acid | 1081.2(828.6,1436.8) | 1137.9(846.7,1508.2) | 1444.2(914.1,1671.4) | <0.001 |
| Hippuric acid | 438.2(255.6,1244.9) | 561.7(282.8,1656.2) | 2353.0(334.7,3027.2) | <0.001 |
| Homogentisic acid | 19.8 (9.6,20.1) | 19.7 (7.5,20.1) | 4.2(3.4,9.1) | <0.001 |
| Citric acid | 15918.1(11645.3,19924.2) | 16296.0(12481.5,20110.2) | 20346.3 (16013.5,25906.8) | <0.001 |
| Malic acid | 781.0 (593.7,1099.0) | 817.5 (621.4,1140.1) | 1016.7 (762.6,1455.0) | <0.001 |
| Glucaric acid | 64.0(47.7,87.7) | 64.3(46.7,91.1) | 57.5(37.2,137.3) | 0.215 |
| Orotic acid | 99.9(82.8,112.4) | 99.7(82.1,113.3) | 64.3(43.4,113.3) | <0.001 |
| L-Lactic acid | 348655.4(259263.6,654447.3) | 382600.2(271296.0,635277.3) | 493482.0 (362988.5,909554.6) | <0.001 |
| Maleic acid | 85.3(58.1,130.1) | 91.7(59.9,139.4) | 80.9(43.5,132.1) | 0.011 |
| cis-Aconite acid | 350.9(250.0,470.5) | 360.8(249.4,490.8) | 469.5(330.7,720.3) | <0.001 |
| Isocitric acid | 3516.0(1981.5,4837.2) | 3588.2(2072.5,5150.7) | 4501.6(2801.4,6560.7) | <0.001 |

* P-value is derived from the Kruskal-Wallis H test.

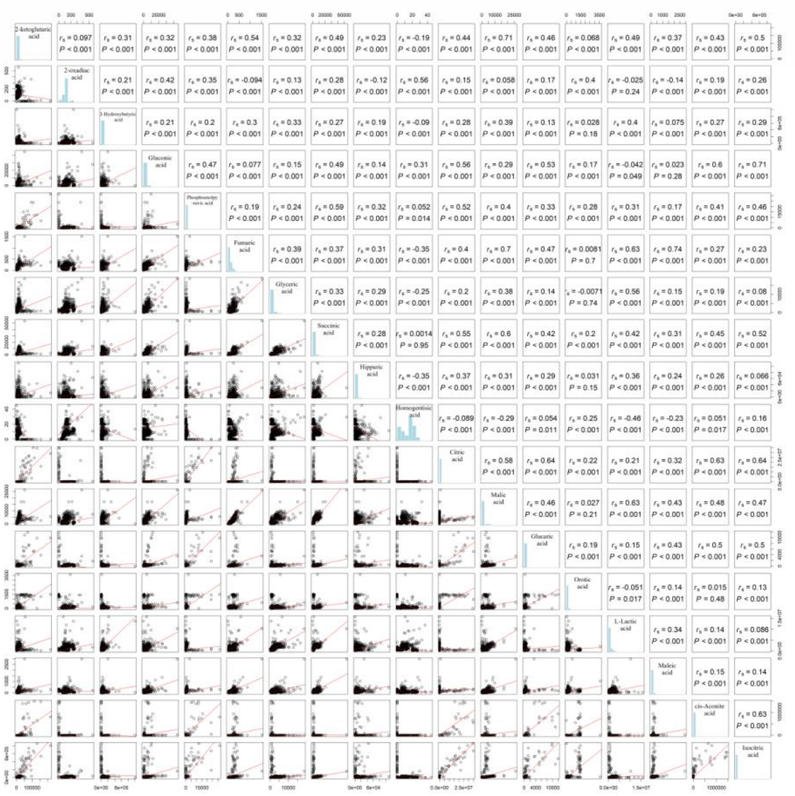

**Fig.S2.** Spearman correlation coefficient map of 18 central carbon metabolites

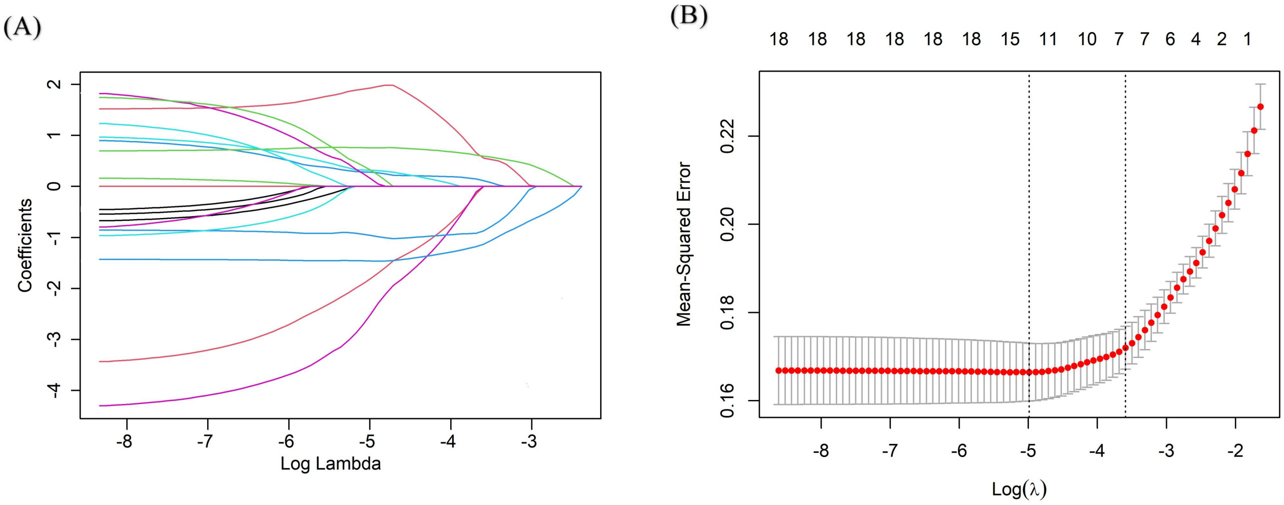


**Fig.S3.** LASSO regression selects metabolites of model PPNs risk prediction model in high-risk lung cancer population. (A) The change trajectory of each variable coefficient (λ). (B) Cross-validation plot for the penalty term

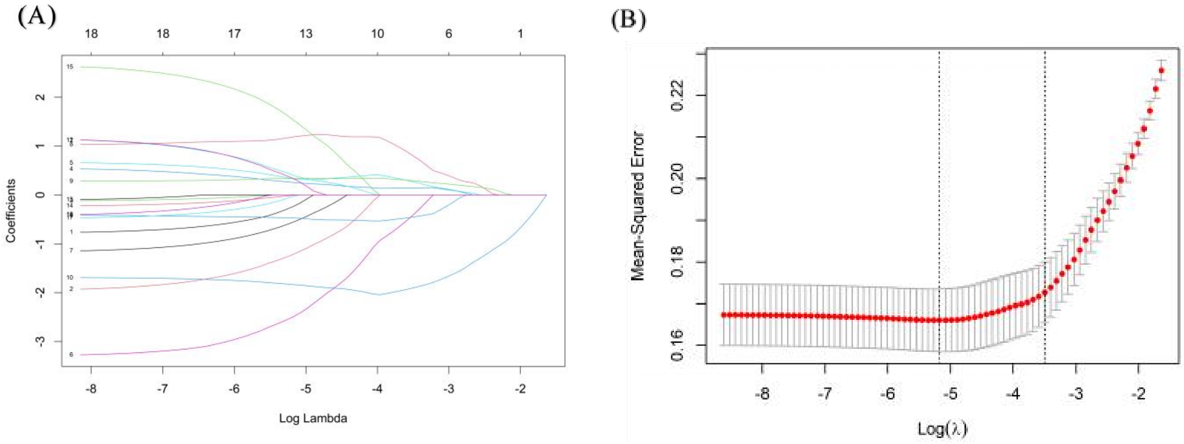


**Fig.S4.** LASSO regression selects metabolites of PPNs versus NPNs. (A) The change trajectory of each variable coefficient (λ). (B) Cross-validation plot for the penalty term

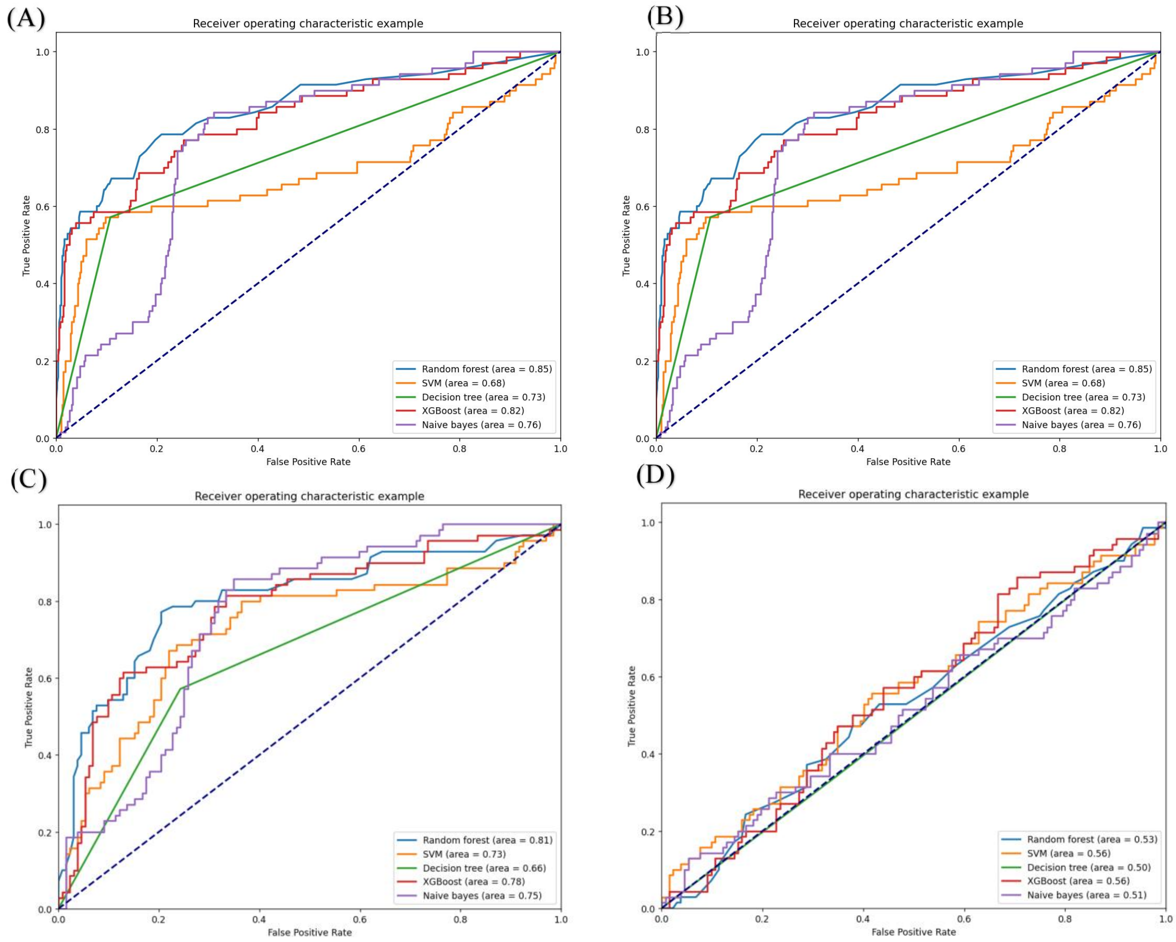


**Fig. S5.** ROC curves of prediction models in different data sets. (A) PPNs risk prediction model in high-risk lung cancer population (metabolite-only) (B) PPNs risk prediction model in high-risk lung cancer population (demographic characteristics and clinical general detection indicators) (C) PPNs versus NPNs (metabolite-only) (D) PPNs versus NPNs (demographic characteristics and clinical general detection indicators). XGBoost: Extreme Gradient Boosting; SVM: support vector machine
